# Correction to: miR-195-5p/NOTCH2-mediated EMT modulates IL-4 secretion in colorectal cancer to affect M2-like TAM polarization

**DOI:** 10.1186/s13045-019-0810-x

**Published:** 2019-11-22

**Authors:** Xiaobin Lin, Shuyi Wang, Min Sun, Chunxiao Zhang, Chen Wei, Chaogang Yang, Rongzhang Dou, Qing Liu, Bin Xiong

**Affiliations:** 1 Department of Gastrointestinal Surgery, Zhongnan Hospital of Wuhan University, No.169 Donghu Road, Wuchang District, Wuhan, 430071 China; 2 Department of Gastric and Colorectal Surgical Oncology, Zhongnan Hospital of Wuhan University, No.169 Donghu Road, Wuchang District, Wuhan, 430071 China; 3 Hubei Key Laboratory of Tumor Biological Behaviors, No.169 Donghu Road, Wuchang District, Wuhan, 430071 China; 4 Hubei Cancer Clinical Study Center, No.169 Donghu Road, Wuchang District, Wuhan, 430071 China; 5 Department of General Surgery, Taihe Hospital, Hubei University of Medicine, Shiyan, 442000 China

**Correction to: J Hematol Oncol (2019) 12:20**

**https://doi.org/10.1186/s13045-019-0708-7**

After publication of this article [1], we were alerted to the inappropriate use of the term "ISET device." ISET is a trademarked product, which was not used in this study. For clarification, we would like to state that where the article refers to “ISET,” it should instead read “CTCBIOPSY® (Wuhan YZY Medical Science and Technology Co., Ltd., Wuhan, China).”
